# Association between subclinical left ventricular ejection fraction and platelet-to-lymphocyte ratio in patients with peritoneal dialysis

**DOI:** 10.3389/fmed.2022.961453

**Published:** 2022-12-01

**Authors:** Yingjie Duan, Zhong Peng, Shuzhu Zhong, Hong Huang, Zhangxiu He

**Affiliations:** ^1^Department of Nephrology, Hengyang Medical School, The First Affiliated Hospital, University of South China, Hengyang, Hunan, China; ^2^Department of Gastroenterology, Hengyang Medical School, The First Affiliated Hospital, University of South China, Hengyang, Hunan, China; ^3^Hengyang Medical School, Institute of Clinical Medicine, The First Affiliated Hospital, University of South China, Hengyang, Hunan, China

**Keywords:** platelet-to-lymphocyte (PLR), left ventricular ejection function (LVEF), peritoneal dialysis, neutrophil-to-lymphocyte ratio (NLR), monocyte-to-lymphocyte ratio (MLR)

## Abstract

**Background:**

Reduced left ventricular ejection function (LVEF) was associated with increased mortality in patients with peritoneal dialysis (PD) in Asia and the United States of America. The neutrophil-to-lymphocyte ratio (NLR) and platelet-to-lymphocyte ratio (PLR) were correlated with LVEF in PD. However, little information is available regarding the relationship between monocyte-to-lymphocyte ratio (MLR), left ventricular ejection fraction (LVEF), and the use of NLR, PLR, and MLR in predicting left ventricular systolic dysfunction (LVSD) in patients with PD.

**Methods:**

All 181 patients with PD were enrolled between 2014 and 2021 from the Nephrology Department of the First Affiliated Hospital of the University of South China. Demographic features, clinical characteristics, laboratory values, and echocardiographic parameters were collected.

**Results:**

The mean age of patients with PD was 47.4 ± 12.6, and 90 (49.7%) of the patients were men. LVEF showed a negative correlation with PLR (*r* = −0.200, *p* = 0.007) and MLR (*r* = −0.146, *p* = 0.049). The levels of NLR, PLR, and MLR were elevated in patients with PD with LVSD compared with those without (all *p* < 0.05). PLR (OR 4.331, 95% CI: 1.223, 15.342) and albumin (OR 13.346, 95% CI: 3.928, 45.346) were significantly associated with LVSD patients with PD in the multivariate logistic analysis. For differentiating patients with PD with LVSD, optimal cutoffs of NLR, PLR, MLR, and albumin were 4.5 (sensitivity: 76.7%, specificity: 55.0%, and overall accuracy: 58%), 202.6 (sensitivity: 66.7%, specificity: 69.5%, and overall accuracy: 69%), 0.483 (sensitivity: 53.3%, specificity: 72.8%, and overall accuracy: 30%), and 34.6 (sensitivity: 72.2%), respectively.

**Conclusions:**

Our results revealed that PLR was better than NLR, MLR, and albumin in predicting LVSD in PD.

## Background

Peritoneal dialysis (PD) is considered to be a vital method for renal replacement therapy in end-stage renal disease (ESRD), and cardiovascular disease (CVD) events are the leading causes of death in patients with PD (1–3). Several studies revealed that reduced left ventricular ejection function (LVEF) is significantly associated with increased all-cause and cardiovascular mortality in patients with PD (3–7). Therefore, changes in left ventricular function, such as LVEF, are essential for patients with PD.

Inflammation is a well-recognized risk factor contributing to excessive cardiovascular mortality in patients with PD (8, 9). The neutrophil-to-lymphocyte ratio (NLR) and platelet-to-lymphocyte ratio (PLR) have recently emerged as inflammatory biomarkers in patients with ESRD. NLR and PLR were reported to be associated with inflammation and can estimate survival in hemodialysis or patients with PD (9–13). Recently, some studies explored the association among NLR, PLR, and LVEF in patients with PD (14). However, there is no information about the relationship between monocyte-to-lymphocyte ratio (MLR) and LVEF in patients with PD.

Inflammation and left ventricular hypertrophy are interrelated and can contribute to cardiovascular morbidity and mortality rates among patients with PD. The effects of these factors can be reflected in low LVEF (15). LVEF is associated with inflammation in patients with PD (14). Moreover, high NLR and PLR were shown to be strong and independent predictors of left ventricular systolic dysfunction (LVSD) in patients with non-ST elevated acute coronary syndrome (16, 17). Nevertheless, the utility of NLR, PLR, and MLR in predicting LVSD remains unknown in patients with PD.

Thus, our study conducted a cross-sectional analysis to evaluate the role of NLR, PLR, and MLR in predicting LVSD in patients with PD. This study aimed to collect data on LVSD through early detection, opening the door for clinical intervention that may slow the progression of cardiac dysfunction.

## Methods

### Population

In this study, our cohort was recruited from inpatients administered between 2014 and 2021 at the Nephrology Department of the First Affiliated Hospital of the University of South China. Our patients with PD used only glucose-based PD solutions, which generally contained 1.5 or 2.5% dextrose. A 4.25% glucose-based PD solution may be used in the short term if the patient has severe hypervolemia. Our enrolled patients were at least 18 years of age, and they were under treatment for at least the past 3 months. The exclusion criteria were as follows: patients who had PD treatment ≤ 2 weeks or had a history of infection 3 months prior, heart failure or pulmonary embolism, acute coronary syndrome, congenital heart disease, valvular heart disease, chronic lung and liver diseases, systemic lupus erythematosus, and scleroderma (12, 14). Of the 243 patients who met the inclusion criteria, 62 met the exclusion criteria or lacked clinical data, resulting in 181 patients being enrolled in the study. These data were validated by the physicians participating in our protocol. The study protocol was designed in accordance with the Declaration of Helsinki and was approved by the Institutional Ethics Committee of the First Affiliated Hospital of the University of South China. All patients provided their written, informed consent.

### Data collection

We collected baseline demographic and clinical data, including gender, age, body mass index, underlying diseases (hypertension, coronary artery disease [CAD], diabetes mellitus [DM]), duration of PD therapy (in months), history of smoking, and current medications. CAD and BMI were defined in our previous study (12, 14).

The transthoracic echocardiographic examination was performed in accordance with the recommendations of the American Society of Echocardiography, and LVEF was measured as described before (14, 18, 19). Other parameters were also collected, including left ventricular diameter (LVD), left atrium diameter (LAD), right ventricular diameter (RVD), right atrium diameter (Ras), pulmonary artery diameter (PA), ascending aortic diameter (AAD), inter-ventricular septum dimension (IVSD), and left posterior ventricular wall (LVPWD).

The laboratory parameters were collected within 30 days of the study; these were serum albumin, blood routine examination (neutrophils, lymphocytes, monocytes, platelet counts, hemoglobin levels), calcium, phosphate, intact parathormone [iPTH], C-reactive protein (CRP), and Kt/V. The eGFR was described before (12, 20). NLR was calculated as an absolute neutrophil count and an absolute lymphocyte count, PLR as an absolute platelet count and lymphocyte count, and MLR as an absolute monocyte count and lymphocyte count (12).

### Statistical analysis

All statistical analysis was performed by SPSS software (version 21.0) (SPSS Inc., Chicago, IL, USA). Normally distributed variables were expressed as mean ± standard deviation and compared by Student's *t-*test. Non-normally distributed variables were expressed as medians with interquartile ranges (IQRs) and analyzed by the Mann–Whitney test (12). Fisher's exact test was used for the comparison of qualitative data. Pearson's and Spearman's rank correlation investigated the trend and strength of associations between LVEF and various risk factors.

Significant factors associated with LVSD were identified by the univariate and multivariate logistic regression analyses. Data analysis was initiated with univariate logistic regression analysis to screen for potential candidate variables. Then, these candidates (including NLR, PLR, MLR, and albumin) and potential confounders were input to the same model for multivariate analysis. The final regression model comprised the following variables: age, duration of peritoneal dialysis, diabetes, NLR, PLR, MLR, albumin, serum phosphate, and CRP. The beta coefficient, along with the odds ratio (OR) and 95% confidence interval (CI), was calculated, and the *p-*value < 0.05 was considered significant (12).

Using receiver operating characteristic (ROC) curves, the potential predictive value of NLR, PLR, MLR, and albumin for LVSD in patients with PD was assessed. Based on the optimal cutoffs of NLR, PLR, and MLR from ROC analysis, we used 4.5, 202.6, and 0.483 of NLR, PLR, and MLR for categorizing into two groups: NLR ≤ 4.5 vs. NLR > 4.5, PLR ≤ 202.6 vs. PLR > 202.6, and NLR ≤ 0.483 vs. PLR > 0.483, respectively. A *P-*value of < 0.05 was considered significant.

## Results

### Characteristics of the study subjects

Among 181 patients with PD, the mean age was 47.4 ± 12.6 years, and 90 of them (49.7%) were men. The etiological factors of ESRD were chronic glomerulonephritis in 92 (50.8%), diabetic nephropathy in 20 (11.0%), hypertensive nephropathy in 7 (3.9%), others in 13 (7.2%), and undetermined in 49 (27.1%). The average value of LVEF was 59.4%, and the prevalence of LVSD was 16.0%. Demographic characteristics, clinical features, laboratory values, and echocardiographic parameters were summarized in Table 1.

**Table 1:** Basic information of demographic, laboratory and echocardiographic characteristics in peritoneal dialysis patients.

	**Total (*n =* 181)**	**Neutrophil-to-lymphocyte ratio**		***P***	**Platelet-to-lymphocyte ratio**		***P***	**Monocyte-to-lymphocyte ratio**		***P***
		**≤4.5 (*n =* 91)**	**>4.5 (*n =* 90)**		**≤202.6 (*n =* 115)**	**>202.6 (*n =* 66)**		**≤0.483 (*n =* 124)**	**>0.483 (*n =* 57)**	
**Demographics**										
Age (years)	47.4 ± 12.6	45.5 ± 12.7	48.8 ± 11.8	0.15	45.6 ± 12.3	50.7 ± 12.5	**<0.01**	46.5 ± 13.3	49.4 ± 10.7	0.16
Male, n (%)	90 (49.7)	39 (42.9)	51 (56.7)	0.06	56 (48.7)	34 (51.5)	0.72	56 (45.2)	34 (59.6)	0.07
BMI (kg/m^2^)	22.0 ± 3.5	21.6 ± 3.3	22.6 ± 3.8	0.12	22.3 ± 3.5	21.4 ± 3.5	0.19	21.7 ± 3.4	22.8 ± 3.8	0.12
Diabetes, n (%)	20 (11.0)	7 (7.7)	13 (14.4)	0.15	7 (6.1)	13 (19.7)	**<0.01**	12 (9.7)	8 (14)	0.39
Hypertension, n (%)	168 (92.8)	83 (91.2)	85 (94.4)	0.39	105 (91.3)	63 (95.5)	0.29	113 (91.1)	55 (96.5)	0.19
Previous CAD, n (%)	12 (6.6)	6 (6.6)	6 (6.7)	0.98	5 (4.3)	7 (10.6)	0.10	9 (7.3)	3 (5.3)	0.62
Smoker, n (%)	25 (13.8)	10 (11.0)	15 (16.7)	0.27	14 (12.2)	11 (16.7)	0.39	19 (15.3)	6 (10.5)	**<0.01**
Duration of CAPD (month)	15.0 (3.0, 34.5)	12.0 (3.0, 34.0)	20 (4.5, 36.5)	0.08	12.0 (3.0, 31.0)	23.5 (6.0, 42.8)	**0.02**	12.0 (3.0, 34.0)	23.0 (7.5, 35.5)	**0.04**
Kt/V	1.93 ± 0.63	2.03 ± 0.61	1.79 ± 0.62	0.051	1.93 ± 0.64	1.92 ± 0.62	0.91	1.97 ± 0.62	1.83 ± 0.64	0.30
**Blood biochemistry**										
NLR	4.5 (3.2, 6.6)	3.2 (2.5, 3.9)	6.7 (5.5, 10.6)	**<0.01**	4.0 (2.9, 4.8)	7.28 (5.1, 11.3)	**<0.01**	3.0 (2.8, 4.9)	8.0 (5.5, 10.9)	**<0.01**
PLR	170 (117, 241)	130 (94, 168)	220 (163, 329)	**<0.01**	127 (94, 165)	293 (230, 374)	**<0.01**	139 (104, 200)	222 (179, 379)	**<0.01**
MLR	0.4 (0.3, 0.5)	0.3 (0.2, 0.4)	0.5 (0.4, 0.8)	**<0.01**	0.3 (0.3, 0.4)	0.5 (0.4, 0.8)	**<0.01**	0.3 (0.3, 0.4)	0.7 (0.6, 0.9)	**<0.01**
Hemoglobin (g/L)	76.6 ± 18.9	75.7 ± 15.9	77.5 ± 22.1	0.77	76.2 ± 18.5	77.3 ± 19.9	0.93	76.7 ± 18.5	76.5 ± 20.1	0.97
Creatine (umol/L)	999.5 ± 357.5	972.3 ± 350.3	1,038.9 ± 371.3	0.23	1,015.3 ± 353.6	971.3 ± 365.3	0.43	1,002.1 ± 376.8	993.6 ± 313.1	0.88
GFR (ml/min/1.73m^2)^	5.9 (4.7, 7.3)	5.9 (4.8, 7.4)	5.5 (4.4, 7.3)	0.16	5.8 (4.4, 7.2)	5.9 (4.8, 7.5)	0.43	5.9 (4.5, 7.2)	5.8 (4.8,7.4)	0.49
iPTH (pg/ml)	321 (163, 495)	357 (221, 504)	260 (137, 483)	0.13	337 (208, 471)	282 (133, 512)	0.34	332 (208, 491)	255 (157, 463)	0.32
Serum albumin (g/L)	37.1 ± 6.5	38.1 ± 5.5	36.4 ± 7.4	0.09	38.1 ± 5.7	35.7 ± 7.4	**0.03**	37.6 ± 6.4	36.2 ± 6.8	0.20
Serum calcium (mmol/L)	2.0 ± 0.3	1.9 ± 0.3	2.0 ± 0.3	0.57	1.9 ± 0.3	2.1 ± 0.3	0.09	1.9 ± 0.3	2.0 ± 0.3	0.29
Serum phosphate (mmol/L)	1.9 ± 0.7	1.9 ± 0.6	1.9 ± 0.7	0.95	2.0 ± 0.7	1.7 ± 0.6	**0.01**	1.9 ± 0.7	1.7 ± 0.7	**0.03**
CRP	3.6 (0.9, 18.7)	2.0 (0.8, 7.0)	9.8 (0.9, 31.5)	**<0.01**	2.7 (1.0, 12.6)	4.1 (0.7, 23.0)	0.45	2.0 (0.7, 10.3)	9.5 (2.0, 30.3)	**<0.01**
**Echocardiographic data**										
LVEF (%)	59.4 ± 10.3	60.5 ± 9.3	58.1 ± 11.1	0.14	60.7 ± 8.4	56.6 ± 12.5	**0.02**	60.3 ± 9.3	57.4 ± 12.0	0.08
LVSD, n (%)	29 (16.0)	9 (9.9)	20 (22.2)	**0.02**	11 (9.6)	18 (27.3)	**<0.01**	3 (2.4)	26 (45.6)	**<0.01**
LVd (mm)	50.7 ± 7.5	49.4 ± 6.7	52.3 ± 8.0	**<0.01**	49.7 ± 6.4	52.4 ± 8.9	**0.04**	49.9 ± 7.3	52.3 ± 7.7	**0.04**
LAs (mm)	34.5 ± 7.3	33.4 ± 6.8	35.9 ± 7.8	0.03	33.9 ± 6.8	35.5 ± 8.1	0.32	33.8 ± 6.9	36.1 ± 8.1	0.05
RVd (mm)	28.6 ± 4.5	28.3 ± 4.3	29.2 ± 4.9	0.22	28.3 ± 3.8	29.4 ± 5.6	0.41	28.7 ± 4.5	28.8 ± 4.7	0.82
RAs (mm)	32.6 ± 5.1	32.6 ± 5.3	32.9 ± 5.2	0.52	32.5 ± 5.0	32.9 ± 5.5	0.68	32.3 ± 5.3	33.6 ± 4.7	0.13
PA (mm)	22.7 ± 3.6	22.4 ± 3.7	23.0 ± 3.5	0.49	22.0 ± 2.9	23.9 ± 4.4	**0.04**	22.3 ± 3.5	23.5 ± 3.8	0.05
AAO (mm)	31.4 ± 14.5	32.5 ± 20.9	30.7 ± 3.4	0.46	32.3 ± 18.7	30.4 ± 3.8	0.58	31.9 ± 18.1	30.9 ± 3.4	0.69
IVSd (mm)	10.3 ± 1.7	10.2 ± 1.4	10.6 ± 1.9	0.29	10.3 ± 1.7	10.6 ± 1.6	0.2	10.4 ± 1.7	10.4 ± 1.7	0.93
LVPWd (mm)	9.7 ± 2.0	9.6 ± 2.2	9.85 ± 1.9	0.43	9.7 ± 2.2	9.8 ± 1.7	0.7	9.8 ± 2.2	9.6 ± 1.6	0.47

All the bold values are P-value that less than 0.05.

There were 91 patients in the NLR ≤ 4.5 group and 90 in the NLR >4.5 group. Their average LVEF was 60.5 ± 9.3% and 58.1 ± 11.1%, respectively (*P* = 0.14). In the comparison of relevant data between groups, no statistical difference was found in demographic characteristics. Compared with the low NLR group, the high NLR group contained more patients with LVSD (*p* < 0.01), higher LVD (*p* < 0.01), and a higher level of CRP (*p* < 0.01) (Table 1).

There were 115 patients in the PLR ≤ 202.6 group and 66 in the PLR > 202.6 group. Their average LVEF was 60.7 ± 8.4% and 56.6 ± 12.5%, respectively (*P* = 0.02). In comparison with patients with PD in the PLR ≤ 202.6 group, those with PLR >202.6 were older (*p* < 0.01) and had more patients with diabetes (*p* < 0.01) or LVSD (*p* < 0.01). Moreover, patients with PLR >202.6 had a lower level of serum albumin (*p* = 0.03) and serum phosphate (*p* = 0.01) than those with PLR ≤ 202.6 (Table 1).

There were 124 patients with PD in MLR ≤ 0.483 group and 57 in MLR >0.483 group. Their average LVEF was 60.3 ± 9.3 and 57.4 ± 12.0, respectively (*P* = 0.08). The number of smokers in patients in the low MLR group was more than that in the high MLR group (*p* < 0.01). The high MLR group had a larger number of LVSD patients and a higher level of CRP (*p* < 0.01) than the low MLR group (< 0.01). As to other clinical values, no statistical difference was found (Table 1).

### NLR, PLR, and MLR levels and correlations of LVEF with characteristics of patients with PD

**Figure 1:**
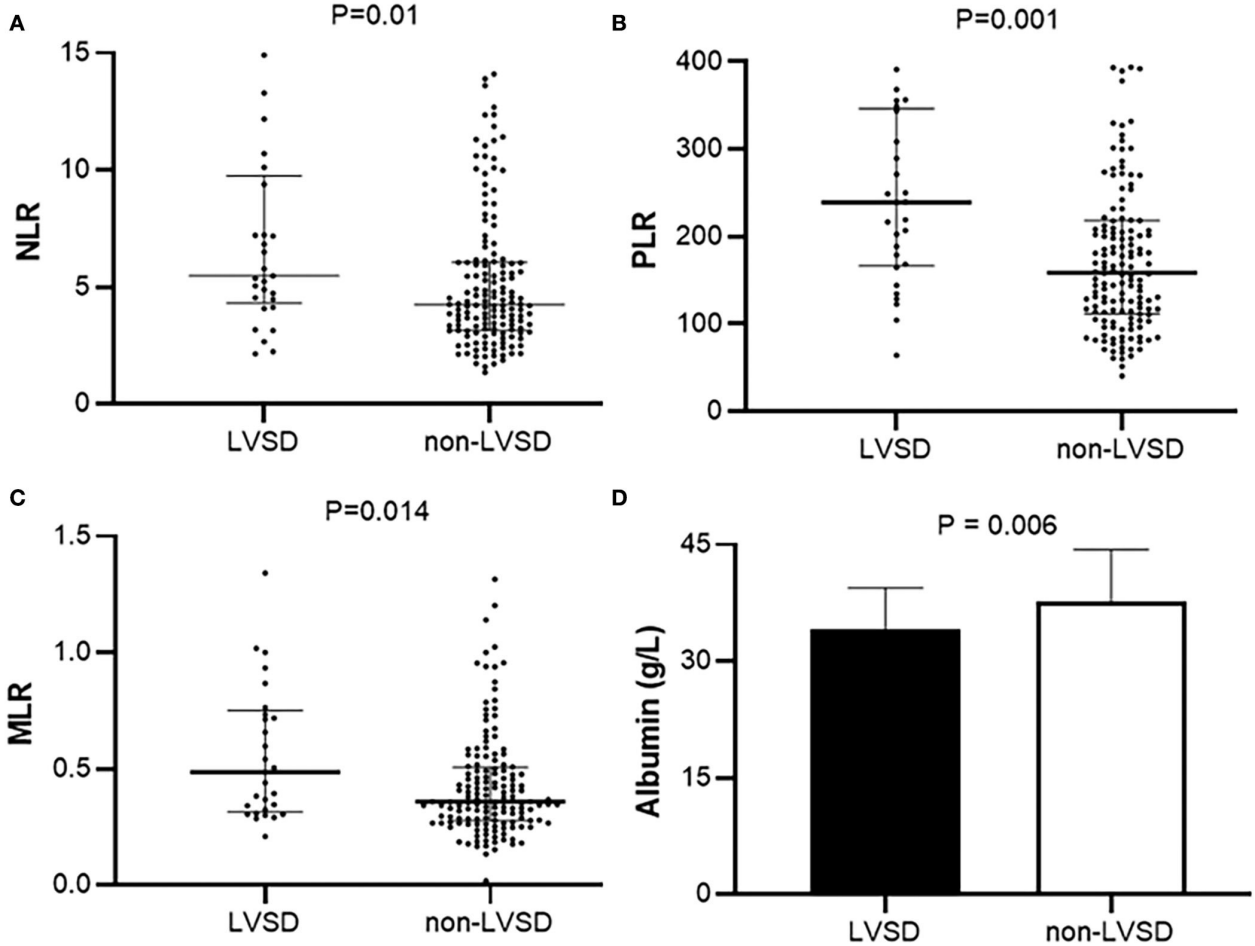
Levels of **(A)** NLR, **(B)** PLR, **(C)** MLR, and **(D)** serum albumin in patients with PD with LVEF < 50% (LVSD) and with LVEF≥50% (non-LVSD) in peritoneal patients. NLR, Neutrophil-to-lymphocyte ratio; PLR, Platelet-to-lymphocyte; MLR, Monocyte-to-lymphocyte ratio; LVSD, Left ventricular systolic dysfunction.

Figure 1 shows the levels of NLR, PLR, and MLR in patients with PD with LVEF < 50% (LVSD) and with LVEF≥50% (non-LVSD). The levels of NLR, PLR, and MLR were increased in patients with PD with LVSD compared with those without [5.5 (4.5, 9.4) vs. 4.2 (3.2, 6.1), *p* = 0.01; 239.1 (168.5, 343.9) vs. 158.9 (112.3, 218.2), *p* = 0.001; 0.5 (0.3, 0.7) vs. 0.4 (0.3–0.5), *p* = 0.014], while albumin was decreased in patients with PD and LVSD compared with that in those without (34.1 ± 5.3 g/L vs. 37.8 ± 6.6g/L, *p* = 0.006).

**Figure 2:**
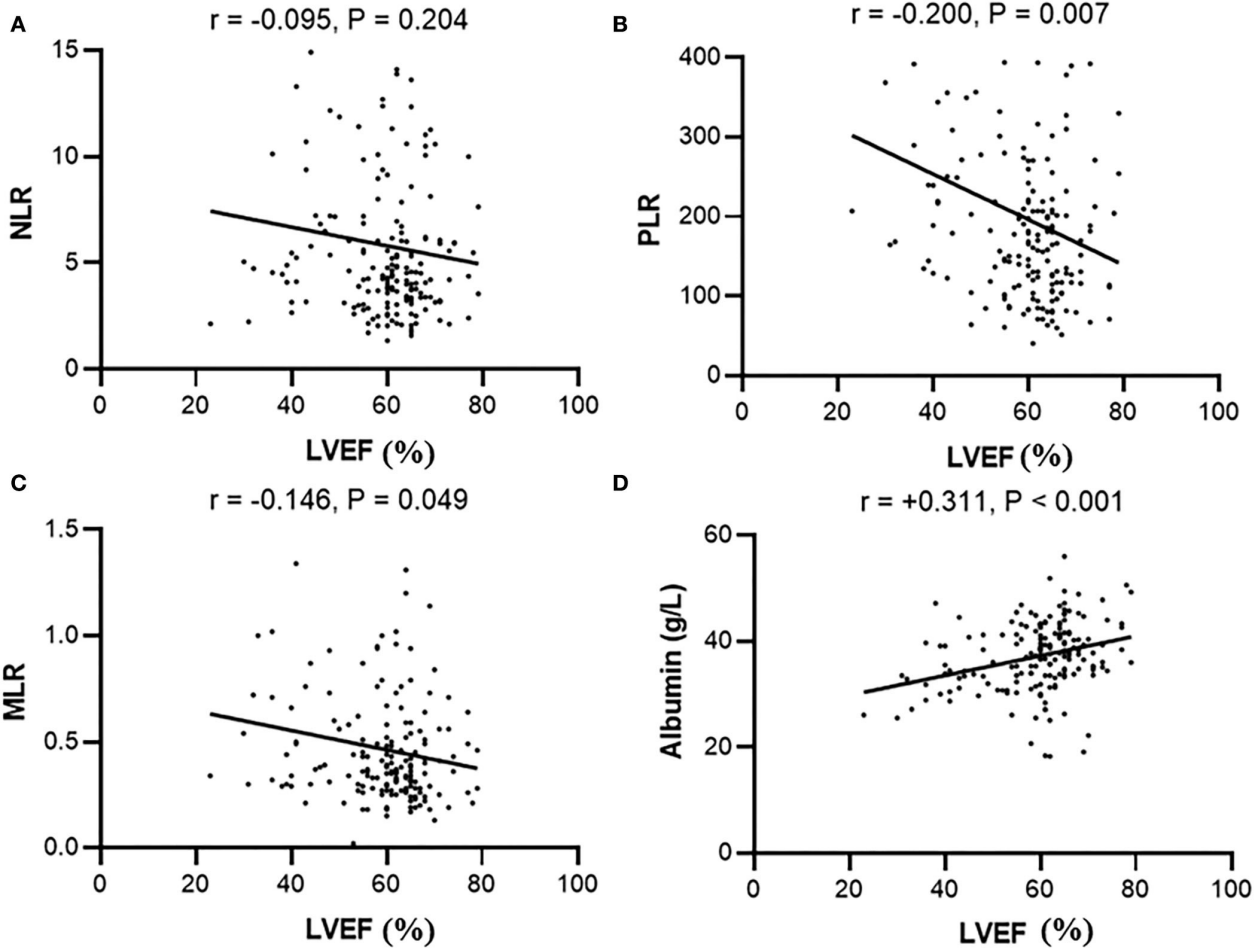
Correlation between LVEF and **(A)** NLR, **(B)** PLR, **(C)** MLR, or **(D)** serum albumin in peritoneal patients. NLR, Neutrophil-to-lymphocyte ratio; PLR, Platelet-to-lymphocyte; MLR, Monocyte-to-lymphocyte ratio.

LVEF showed a negative correlation with PLR (*r* = −0.200, *p* = 0.007), MLR (*r* = −0.146, *p* = 0.049), LVD (*r* = −0.519, *p* < 0.001), LAs (*r* = −0.299, *p* < 0.001), and PA (*r* = −0.161, *p* = 0.038). Meanwhile, LVEF was positively correlated with lymphocytes (*r* = 0.182, *p* = 0.014) and serum albumin (*r* = 0.311, *p* < 0.001) (Table 2). However, we did not find a statistical difference between LVEF and NLR (*r* = −0.095, *p* = 0.204) (Figure 2).

**Table 2:** Correlationship between LVEF and study parameters in peritoneal dialysis patients.

	***r***	***P*-value**
Age (years)	−0.038	0.610
BMI (kg/m^2^)	+0.106	0.261
Duration of CAPD (months)	−0.082	0.274
Kt/V	+0.072	0.458
NLR	−0.095	0.204
PLR	−0.200	**0.007**
MLR	−0.146	**0.049**
Lymphocyte	+0.182	**0.014**
Hemoglobin (g/L)	+0.013	0.860
eGFR (ml/min/1.73m^2^)	−0.035	0.646
iPTH (pg/ml)	+0.007	0.927
Serum albumin (g/L)	+0.311	**<0.001**
Serum calcium (mmol/L)	+0.081	0.285
Serum phosphate (mmol/L)	+0.064	0.405
CRP	−0.147	0.079
LVd (mm)	−0.519	**<0.001**
LAs (mm)	−0.299	**<0.001**
RVd (mm)	−0.127	0.089
RAs (mm)	−0.080	0.287
PA (mm)	−0.161	**0.038**
AAO (mm)	−0.006	0.936
IVSd (mm)	−0.110	0.140
LVPWd (mm)	−0.170	0.022

All the bold values are P-value that less than 0.05.

### Risk factors for LVSD in patients with PD

We applied univariate and multivariate logistic regression analysis to identify the relationship between LVSD and associated factors. The LVSD was found to be associated with NLR>4.5, PLR >202.6, PLR >0.483, and albumin ≤ 34.6 (Table 3, all *p* < 0.05). However, the multivariate logistic analysis only showed that PLR (OR 4.331, 95% CI: 1.223, 15.342) and albumin (OR 13.346, 95% CI: 3.928, 45.346) were significantly associated with LVSD (Table 3).

**Table 3:** Factors associated with LVSD (LVEF<50%): Univariate and multivariate analysis among peritoneal dialysis patients.

**Factors**	**Univariate analysis**			**Multivariate analysis**		
	**Odds Ratio**	**95%CI**	***P*-value**	**Odds Ratio**	**95%CI**	***P*-value**
Age	0.981	0.95, 1.013	0.246	0.996	0.948, 1.046	0.865
Duration	0.989	0.97, 1.009	0.286	0.990	0.960, 1.020	0.494
Previous CAD	1.007	0.209, 4.848	0.993			
Diabetes	1.137	0.356, 3.634	0.829	8.264	0.833, 82.006	0.071
Smoker	1.737	0.629, 4.796	0.287			
Kt/V	0.566	0.224, 1.429	0.229			
NLR >4.5	3.192	1.322, 7.710	**0.010**	1.078	0.287, 4.051	0.912
PLR >202.6	4.245	1.834, 9.827	**0.001**	4.331	1.223, 15.342	**0.023**
MLR >0.48	2.806	1.248, 6.311	**0.013**	2.478	0.695, 8.836	0.162
Albumin ≤ 34.6 (g/L)	3.056	1.271, 7.348	**0.013**	13.346	3.928, 45.346	**<** **0.001**
Hemoglobin >76.6 (g/L)	1.000	0.456, 2.194	1.000			
Calcium >2.0 (mmol/L)	0.816	0.343, 1.939	0.645			
Phosphate >1.9 (mmol/L)	1.004	0.434, 2.327	0.992	1.163	0.348, 3.886	0.806
CRP >1.445	0.667	0.078, 5.673	0.711	0.989	0.061, 16.093	0.982

All the bold values are P-value that less than 0.05.

### ROC curves of NLR, PLR, MLR, and albumin for predicting LVSD

**Figure 3:**
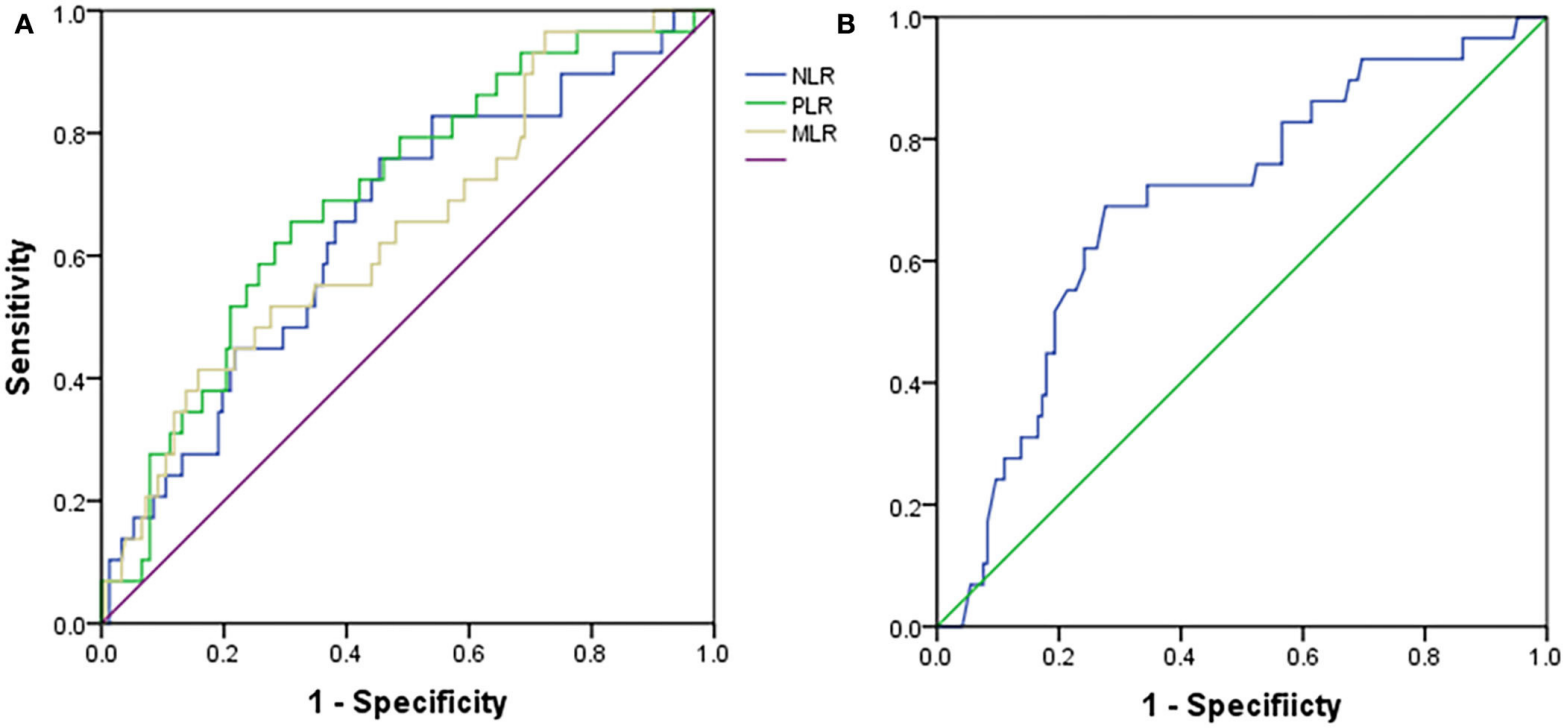
Receiver operating characteristic curves for **(A)** NLR, PLR, MLR, and **(B)** albumin in peritoneal dialysis patients with LVSD or without LVSD. NLR, Neutrophil-to-lymphocyte ratio; PLR, Platelet-to-lymphocyte; MLR, Monocyte-to-lymphocyte ratio; LVSD, Left ventricular systolic dysfunction.

The ROC analyses were used to establish the NLR, PLR, MLR, and albumin cutoff points. For differentiating patients with PD with LVSD, optimal cutoffs of NLR, PLR, MLR, and albumin based on the largest Youden index were 4.5 (sensitivity: 76.7%, specificity: 55.0%, and overall accuracy: 58%), 202.6 (sensitivity: 66.7%, specificity: 69.5%, and overall accuracy: 69%), 0.483 (sensitivity: 53.3%, specificity: 72.8%, and overall accuracy: 30%), and 34.6 (sensitivity: 72.2%, specificity: 66.7%, and overall accuracy). The results suggested that NLR (area under the curve (AUC): 0.664), PLR (AUC: 0.705), MLR (AUC: 0.651), and albumin (AUC: 0.692) might be useful for distinguishing between patients with PD with LVSD and non-LVSD (Figure 3 and Table 4).

**Table 4:** The statistical values of NLR, PLR, MLR, and albumin in prediction of LVSD in peritoneal dialysis patients.

	**AUC**	**95%CI**	**Sensitivity (%)**	**Specificity (%)**	***P*-value**	**Overall accuracy**
NLR (cutoff: >4.5)	0.664	0.557–0.771	76.7	55.0	**0.005**	0.58
PLR (cutoff: >202.6)	0.705	0.607–0.804	66.7	69.5	**0.000**	0.69
MLR (cutoff: >0.483)	0.651	0.545–0.758	53.3	72.8	**0.009**	0.30
Albumin (cutoff: ≤ 34.6)	0.692	0.592–0.793	72.2	66.7	**0.001**	0.69

All the bold values are P-value that less than 0.05.

## Discussion

In this study, we enrolled 181 patients with PD, with or without LVSD. As it turned out, NLR, PLR, and MLR levels were all shown to be higher in patients with PD and LVSD than in those without LVSD. Our analysis showed that PLR and MLR were significantly negatively associated with LVEF, while lymphocytes and serum albumin were significantly positively associated with LVEF in patients with PD. Further multivariate logistic analysis indicated that PLR >202.6 or serum albumin ≤ 34.6 g/L were risk factors for LVSD in patients with PD. Additionally, our results revealed that PLR was better than NLR, MLR, and albumin in predicting LVSD in patients with PD.

The prevalence of LVSD in the dialysis population is much greater than in the general population (3, 21, 22). The prevalence of LVSD in our CA patients with PD was 16%, similar to the Thai CAPD population (17.48%) (14). They are much larger than in incident CA patients with PD (6.6%) (3). A study of stable patients with PD and LVSD indicated that the odds ratios for death were 1.93 (3). LVSD in patients with PD can partially be attributed to factors such as a chronic inflammatory state, uremia toxin, anemia, fluid retention, hyperparathyroidism, renin-angiotensin-aldosterone activation, an increased serum calcium-phosphate product, and glucose load (23). Although LVEF was significantly negatively related to LVD, LAs, and PA in the current study, those data needed to be obtained from transthoracic echocardiographic parameters. Since the subjectivity of transthoracic echocardiographic examination is especially high in rural districts with poor medical resources, we wondered whether there were other parameters readily available from routine laboratory tests that could be used to predict the risk of developing left ventricular malformations in patients with PD.

Peritoneal patients are in a chronic and persistent inflammation status where leukocytes such as neutrophils, lymphocytes, and monocytes play an important role (8). Previous literature has shown that inflammatory cytokines released by leukocytes promote myocardial cell apoptosis and fibrosis, leading to the progression of heart failure and left ventricular remodeling (24). Thus, we decided to explore the relationship among NLR, PLR, MLR, and LVEF. Neutrophil activation occurs during uremia or heart failure, potentially contributing to inflammation in those patients (25, 26). Indeed, accelerated programmed cell death and apoptosis have been observed in lymphocytes among ESRD patients, leading to a decreased level of lymphocytes in our current study (27). Therefore, it is reasonable to understand our result that the levels of NLR, PLR, and MLR were elevated in patients with PD and LVSD compared with the non-LVSD group.

Additionally, MLR was negatively related to LVEF. This is supported by the report that higher MLR levels may be the independent factor associated with increased CVD mortality in patients with PD (28). We first addressed the association between MLR and LVSD in patients with PD. Previous studies demonstrated that MLR or NLR was mainly used to predict inflammation (12, 29), and their value in evaluating LVSD remains to be explored by further study.

In the current study, our univariate analysis demonstrated that NLR, PLR, MLR, and albumin might be risk factors for LVSD in patients with PD. Recently, a published report showed that NLR, PLR, hemoglobin, serum calcium, and phosphate levels might be conducive to modifying the risk of LVSD in patients with PD (14). With 181 PD patients recruited in the current study compared to 103 in the study by Angkananard et al., we had a larger sample size, which is a strength of our research (14). Another difference is that our Chinese patients had a longer median duration of CAPD (15 months) than that of Thai patients (13 months) (14). A different number of patients with PD, duration of CAPD time, and race may all have contributed to different results in different studies. PLR could be regarded as a significant independent predictor of long-term mortality after a non-ST segment elevation myocardial infarction (30). A high PLR is a strong and independent predictor for LVSD in patients with non-ST elevated acute coronary syndrome (16). Consistent with this, our results revealed that PLR >202.6 was still a risk factor (OR: 4.331) for LVSD in patients with PD, even in multivariate analysis. Nevertheless, the usefulness of PLR in predicting LVSD in patients with PD has not been determined. In this study, our ROC analysis suggested that PLR (AUC: 0.705) was better than NLR (AUC: 0.664), MLR (AUC: 0.651), and albumin (AUC: 0.692) and might be an additional diagnostic tool in disguised LVSD from non-LVSD patients with PD. Previous research demonstrated that reduced LVEF is significantly associated with increased all-cause and cardiovascular mortality in patients with PD. PLR was independently associated with all-cause mortality in patients with PD (3, 31). This implies that PLR may have its own special usefulness in identifying LVSD in patients with PD, and its potential value still needs to be explored in the future.

Serum albumin is also an important inflammatory marker. Low baseline serum albumin levels can reliably predict mortality in patients with PD (32). Consistent with this, our results showed that serum albumin was significantly positively associated with LVEF, and serum albumin ≤ 34.6 g/L was a risk factor for LVSD in our patients with PD. We then explored the ability of albumin to distinguish LVSD from non-LVSD patients with PD. Surprisingly, we discovered that serum albumin has the same overall accuracy (0.69) with a sensitivity of 72.2% and specificity of 66.7% as PLR, although its AUC (0.692) was slightly smaller than that of PLR (0.705). However, in our univariate and multivariate analyses, CRP was not statistically related to LVEF and was not a risk factor for LVSD in peritoneal patients. This implied that the ability of NLR/PLR/MLR and albumin to predict LVSD in peritoneal patients might have its own special or potential value beyond the traditional inflammation.

This study has some limitations: First, it was a cross-sectional and single-center study, and the causal relationship of LVSD among various variables could not be determined in patients with PD. Secondly, the data are based on a single measurement in the laboratory. In the future, a dynamic relationship between LVSD and the related blood parameters will be needed. Inflammatory markers such as hypersensitive CRP and procalcitonin (PCT), considered expensive, were not included in our analysis. Therefore, further longitudinal studies, including more participants and centers, are needed to explore the observational relationship between different dialysis modes and long-term survival in LVSD patients with PD.

## Conclusion

PLR, as an inexpensive and easily calculable marker, is an independent predictor of LVSD in patients with PD. The predictive capacity of PLR is superior to that of NLR, MLR, and albumin, which are other well-known inflammatory markers. The potential value of PLR still needs to be explored in patients with PD in the future.

## Data availability statement

The original contributions presented in the study are included in the article/supplementary material, further inquiries can be directed to the corresponding author/s.

## Ethics statement

The study protocol was approved by the Institutional Ethics Committee of the First Affiliated Hospital of University of South China. All patients gave their written, informed consent to participate in this study.

## Author contributions

Study concept: ZH. Study design, statistical analysis, data interpretation, and manuscript drafting: ZH, YD, and ZP. Data acquisition: YD, ZP, HH, and SZ. All authors contributed to the article and approved the submitted version.

## Funding

This work was supported by the National Natural Science Foundation of China (81900678), the Natural Science Foundation of Hunan Province (2021JJ40491), the Scientific Research Project of the Hunan Health Committee (202103051523), and the Clinical Research Project of the University of South China (USCKF201902G03).

## Conflict of interest

The authors declare that the research was conducted in the absence of any commercial or financial relationships that could be construed as a potential conflict of interest.

## Publisher's note

All claims expressed in this article are solely those of the authors and do not necessarily represent those of their affiliated organizations, or those of the publisher, the editors and the reviewers. Any product that may be evaluated in this article, or claim that may be made by its manufacturer, is not guaranteed or endorsed by the publisher.

## Abbreviations

PD: Peritoneal dialysis
ESRD: End-stage renal disease
CVD: Cardiovascular disease
LVEF: Left ventricular ejection function
NLR: Neutrophil-to-lymphocyte ratio
PLR: Platelet-to-lymphocyte
MLR: Monocyte-to-lymphocyte ratio
LVSD: Left ventricular systolic dysfunction
BMI: Body mass index
CAD: Coronary artery disease
DM: Diabetes mellitus
LVD: Left ventricular diameter
LAD: Left atrium diameter
RVD: Right ventricular diameter
RAD: Right atrium diameter
PAD: Pulmonary artery diameter
AAD: Aorta ascendens diameter
IVSD: Inter-ventricular septum dimension
LVPWD: Left ventricular posterior wall
iPTH: Intact parathormone
AUC: Area under the curve
OR: Odds ratios.
